# ﻿Description of three new species and new distributional data for three species of *Homalotylus* (Hymenoptera, Encyrtidae) from China

**DOI:** 10.3897/zookeys.1184.113292

**Published:** 2023-11-21

**Authors:** Guohao Zu, Hao Xue, Haiyang Wang, Wenquan Zhen, Dawei Huang

**Affiliations:** 1 College of Life Sciences, Nankai University, Tianjin, 300071, China Nankai University Tianjin China; 2 College of Horticulture and Landscape, Tianjin Agricultural University, Tianjin, 300384, China Tianjin Agricultural University Tianjin China; 3 Ocean College, Tangshan Normal University, Tangshan, Hebei, 063000, China Tangshan Normal University Hebei China

**Keywords:** Chalcidoidea, Echthroplexiellini, Encyrtinae, identification key, new species, taxonomy

## Abstract

*Homalotylustianjinensis* Zu, **sp. nov.**, *H.bicolor* Zu, **sp. nov.** and *H.guangxiensis* Zu, **sp. nov.** are described as new to science; *H.agarwali* Anis & Hayat, 1998, *H.hemipterinus* (De Stefani, 1898) and *H.varicolorus* Krishnachaitanya & Manickavasagam, 2016 are newly recorded from China. A key to Chinese species based on females is also presented.

## ﻿Introduction

*Homalotylus* is a well-known genus of Encyrtidae, which was established by Mayr (1876) based on the type species *Encyrtusflaminius* Dalman. Depending on the classification, this genus is placed either in the tribe Homalotylini, subtribe Homalotylina (Trjapitzin 1989) or the tribe Echthroplexiellini, subtribe Homalotylina (Noyes 2010). The species in this genus are solitary or gregarious larval parasitoids, emerging from the prepupal stage of coccinellids (Coleoptera: Coccinellidae) and feeding on sternorrhyncha hemipterans (Hemiptera: Aphidoidea, Coccoidea, Psylloidea). Records of scale insects as hosts are incorrect and those of other hosts, e.g. Chrysomelidae, Bruchidae or Gelechiidae are also probably erroneous (Noyes 2010). The genus contains 66 valid species (Noyes 2019) and nine species from China (Xu and He 1997; Tan and Zhao 1997).

Contributions to the taxonomy of this genus have been made by several authors, such as Trjapitzin (1989) from the Palaearctic, Anis and Hayat (1998) and Krishnachaitanya et al. (2016) from India, Trjapitzin and Ruíz Cancino (1998) from the New World and Xu and He (1997) from China. In this paper, we describe three new species and three new Chinese records of *Homalotylus* from Tianjin and Guangxi, China, and provide a key to the females of the Chinese species.

## ﻿Materials and methods

All the specimens in the present study were collected from the campuses of Tianjin Agricultural University and Beibu Gulf University by malaise traps, then dissected and mounted in Canada Balsam on slides following the method described by Noyes (1982). Morphological terminology and abbreviations follow those of Noyes (2010) with some modifications. Photographs were taken with a digital CCD camera attached to an Olympus BX51 compound microscope. Body lengths were measured using a Leica M205A stereomicroscope; other measurements are relative and taken from micrometer divisions using the eye piece of a stereozoom microscope for carded specimens and the eye piece of a compound microscope for slide-mounted parts, and then transformed into absolute lengths. The holotype of the new species is deposited in the insect collections of Tianjin Agricultural University, China.

The following abbreviations are used in the text:

**F1–6** funicle segments 1–6

**AOL** minimum distance between a posterior ocellus and anterior ocellus

**EL** maximum diameter of eye

**FV** minimum width of the frontovertex

**HH** length of head in facial view, excluding mouth parts

**HW** head width measured in facial view

**MS** malar space or minimum distance between eye and mouth margin

**OCL** minimum distance between a posterior ocellus and occipital margin

**OD** longest diameter of an ocellus

**OOL** minimum distance between a posterior ocellus and eye margin

**POL** minimum distance between posterior ocelli

**FWL** maximum length of fore wing excluding marginal fringe

**FWW** maximum width of fore wing excluding marginal fringe

**MV** marginal vein

**PMV** postmarginal vein

**SMV** submarginal vein

**SV** stigmal vein

**HWL** length of hind wing excluding marginal fringe

**HWW** width of hind wing, measured at widest point, excluding marginal fringe

**MT** length of mid tibia

**GL** length of gonostylus

**LTL** last tergite length

**LTW** last tergite width

**OL** length of ovipositor

**EDAU** Entomology Department, Annamalai University, Chidambaram, India

**HACO** Hayat Collection, Aligarh, India

**USNM** National Museum of Natural History, Washington, D.C., USA

**ZAMU**Zoological Museum, Aligarh Muslim University, Uttar Pradesh, India.

## ﻿Results

### ﻿Key to Chinese species of *Homalotylus* (females)

**Table:** 

1	Ovipositor distinctly exserted, the exserted part at least 0.2× as long as gaster	**2**
–	Ovipositor not or slightly exserted	**11**
2	Funicle completely dark brown, sometimes F6 mixed with white	**3**
–	Funicle at least always with F6 completely white	**5**
3	F6 completely dark brown	***H.zhaoi***
–	F6 brown mixed with white	**4**
4	Frontovetex 0.25× as wide as head width; fore wing 3.30× as long as width	***H.himalayensis***
–	Frontovetex 0.17× as wide as head width; fore wing 2.78× as long as widt	***H.tianjinensis* sp. nov.**
5	F4–F5 always completely white	**6**
–	F4–F5 at least partially white	**8**
6	F3 white	***H.agarwali***
–	F3 dark brown	**7**
7	Axillae and scutellum pale orange yellow	***H.mundus***
–	Axillae dark brown, scutellum mostly dark brown, but yellowish brown apically	***H.trisubalbus***
8	F4 dark brown, F5 at least partially white	**9**
–	F4 dark brown with apical half white, F5 white	**10**
9	Frontovetex 0.19× as wide as head width; mid tibia spur as long as mid basitarsus	***H.longicaudus***
–	Frontovetex 0.25× as wide as head width; mid tibia spur longer than mid basitarsus	***H.varicolorus***
10	Scape 6× as long as wide; clava unsegmented white	***H.scutellaris***
–	Scape 8.42× as long as wide; clava 3-segmented	***H.bicolor* sp. nov.**
11	F4 white; mid tarsus 1–4 white, with basal half of mid basitarsus brown	***H.guangxiensis* sp. nov.**
–	F4 dark brown or brown; mid tarsus 1–4 white	**12**
12	F5 and F6 white	***H.yunnanensis***
–	F5 dark brown, F6 dark brown or partially white, rarely completely white	**13**
13	Mid tibia spur longer than mid basitarsus	***H.sinensis***
–	Mid tibia spur shorter than or equal to mid basitarsus	**14**
14	Mid tibia spur about 8–10× as long as broad; hind tarsus dark brown	***H.flaminius***
–	Mid tibia spur only about 7× as long as broad; hind tarsus mostly white	***H.hemipterinus***

#### ﻿ Homalotylus bicolor

Taxon classificationAnimaliaHymenopteraEncyrtidae

Zu sp. nov.

C29EAB3B-4CAA-51D6-8923-2BEF9189AC70

https://zoobank.org/80414BF3-89A7-4C8B-A62A-319E45842713

Figs 1–6 , 7–12

##### Type materials.

***Holotype*.** ♀ [on slide], China, Guangxi Province, Qinzhou City, Beibu Gulf University, 21°53'53"N, 108°36'56"E, c. 24 m, 06–26.X.2019, Wen-Quan Zhen, Malaise trapping. ***Paratypes*.** ♀♂ [on slides], China, Guangxi Province, Qinzhou City, Beibu Gulf University, 21°53'53"N, 108°36'56"E, c. 24 m, 06–13.X.2019, Wen-Quan Zhen, Malaise trapping.

##### Description.

**Female.** Holotype. Length, 2.20 mm (excluding ovipositor). Head generally orange yellow, with genae, frontovertex and occiput dark brown. Antennae dark brown, F4 mostly white with lower margin brown, F5, F6 and clava white. Mesosoma dark brown with shallow metallic green sheen, scutellum mostly orange yellow, only with a brown area at base; legs dark brown, except for apical 3/5 of mid tibia, mid tibial spur, mid and hind tarsus 1–4 white. Metasoma dark brown.

**Figures 1–6.**
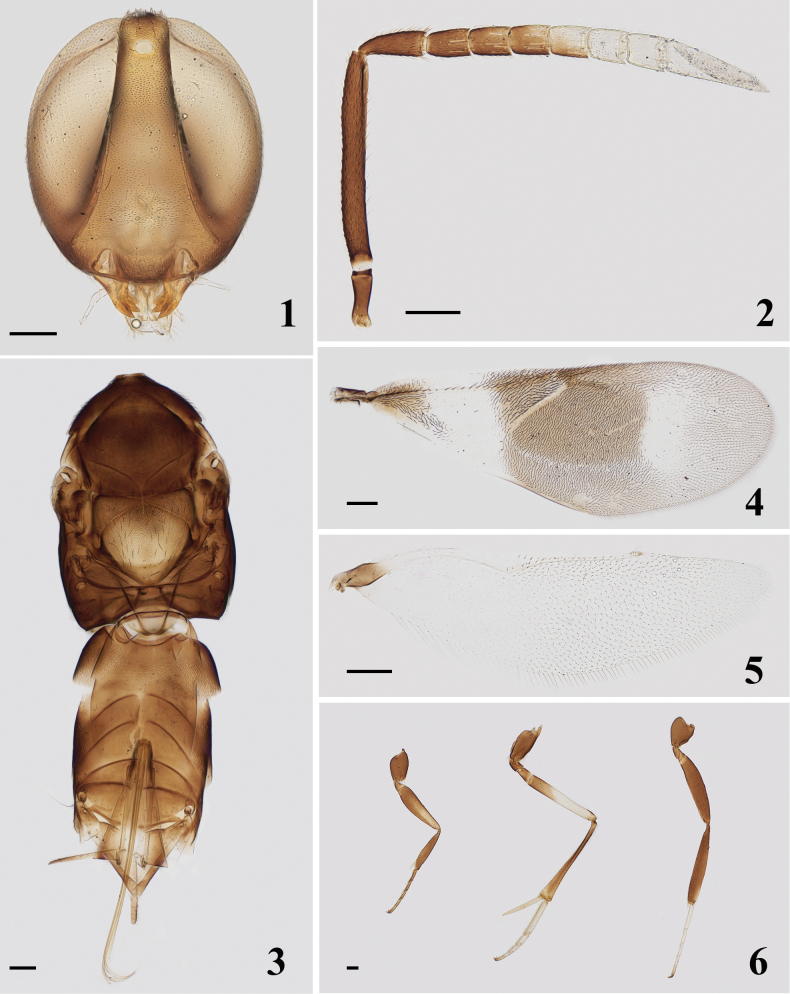
*Homalotylusbicolor* Zu, sp. nov., holotype female **1** head **2** antenna **3** mesosoma and metasoma **4** fore wing **5** hind wing **6** legs. Scale bars: 100 μm.

Head (Fig. 1) with numerous conspicuous setae on frontovertex, each about as long as diameter of posterior ocellus. Head in front view 1.16× higher than wide. Frontovertex 0.22× head width. Ocelli forming an angle of 33°, posterior ocelli close to eye margin, distance to occipital margin 1.50× the diameter of anterior ocellus. Malar space 0.18× eye height. Antennal torulus with its dorsal margin slightly below lower eye margin and very close to oral margin, and the distance of antennal torulus by 1.95× its own height. Antennal (Fig. 2) scape slender, 8.42× as long as wide; pedicel 2.5× as long as wide, 1.5× as long as F1; all funicle segments longer than wide, F1 1.57× as long as wide, F6 1.11× as long as wide; funicle with linear sensilla on all segments; clava 3-segmented, 3.40× as long as wide, and as long as F4–F6 combined, apex strongly obliquely truncate, truncate part approach to the base of the clava. Measurements (μm): HH, 580; HW, 500; FV, 108; EL, 490; MS, 88; OD, 38; POL, 38; OCL, 58; AOL, 78; length and (width)—radicle, 105; scape, 400 (48); pedicel, 125 (50); F1, 83 (53); F2, 70 (55); F3, 75 (58); F4, 73 (60); F5, 70 (63); F6, 70 (63); clava, 213 (63).

Mesosoma (Fig. 3) with sculpture on dorsum similar to that on frontovertex but that on mesoscutum a little shallower; notaular lines conspicuous but not quite meeting at middle of posterior margin of mesoscutum. Mesoscutum 0.72× as long as wide; scutellum 0.94× as long as wide. Fore wing (Fig. 4) 2.65× as long as wide; linea calva interrupted by 4 lines of setae and closed by 5 lines of setae posteriorly; marginal vein 0.44× as long as stigmal vein, and about half the length of postmarginal vein. Hind wing (Fig. 5) 3.73× as long as wide. Length of mid tibial spur (Fig. 6) 0.48× of mid tibia and longer than mid basitarsus. Measurements (μm): FWL, 1325; FWW, 500; SMV, 600; MV, 50; PMV, 100; SV, 113; HWL, 970; HWW, 260; MT, 810; mid tibial spur, 390; mid basitarsus, 360.

Ovipositor (Fig. 3) 1.26× as long as mid tibia, distinctly exserted. Measurements (μm): OL, 1020.

**Male.** Length, 1.67 mm. Color is similar to female, except for F4 completely brown.

**Figures 7–12.**
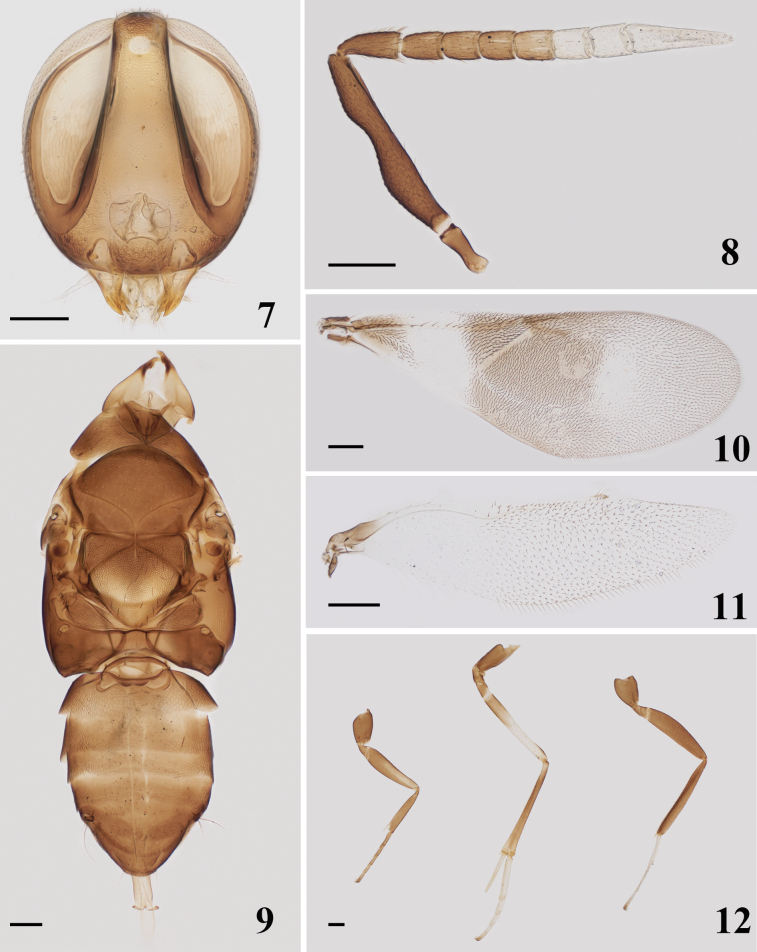
*Homalotylusbicolor* Zu, sp. nov., paratype male **7** head **8** antenna **9** mesosoma and metasoma **10** fore wing **11** hind wing **12** legs. Scale bars: 100 μm.

Head (Fig. 7) in front view 1.11× higher than wide. Frontovertex 0.23× head width, ocelli forming an angle of about 35°. Malar space 0.17× eye height. Antennal torulus with its dorsal margin slightly below lower eye margin and very close to oral margin. Antennal (Fig. 8) scape slender, about 5.9× as long as wide; pedicel 2.18× as long as wide, 1.40× as long as F1; all funicle segments longer than wide; clava unsegmented, 3.72× as long as wide.

Mesoscutum (Fig. 9) 0.58× as long as width; scutellum 0.9× as long as width. Fore wing (Fig. 10) 2.65× as long as width. Hind wing (Fig. 11) 3.86× as long as width. Length of mid tibial spur (Fig. 12) about 0.44× of mid tibia and longer than mid basitarsus.

Aedeagus (Fig. 9) about 1.34× as long as mid tibia.

##### Host.

Unknown.

##### Etymology.

The specific name refers to its coloration of mesosoma.

##### Diagnosis.

The new species may be distinguished from *H.scutellaris* Tan & Zhao, 1997 and *H.mundus* Gahan, 1920, by the following characters: scape 8.42× as long as wide (6× in *H.scutellaris*), clava 3-segmented (unsegmented in *H.scutellaris*), metanotum and propodeum dark brown (yellow brown in *H.scutellaris*); F4 mostly white, mixed with brown on lower margin (completely white in *H.mundus*), axillae brown (pale orange yellow in *H.mundus*), pedicel 2.5× as long as wide (nearly 3× in *H.mundus*).

#### ﻿ Homalotylus guangxiensis

Taxon classificationAnimaliaHymenopteraEncyrtidae

Zu sp. nov.

905DE03F-D65C-5E00-A4A4-2E55F747B79D

https://zoobank.org/7E6B1AC5-27FA-4C9A-93C5-366FF6480BE0

Figs 13–18

##### Type materials.

***Holotype*.** ♀ [on slide], China, Guangxi Province, Qinzhou City, Beibu Gulf University, 21°53'53"N, 108°36'56"E, c. 24 m, 2–11.V.2019, Wen-Quan Zhen, Malaise trapping. ***Paratypes*.** 3♀ [on slides], China, Guangxi Province, Qinzhou City, Beibu Gulf University, 21°53'53"N, 108°36'56"E, c. 24 m, 14–31.XII.2019, 1–13.I.2020, Wen-Quan Zhen, Malaise trapping.

##### Description.

**Female.** Holotype. Length, 2.11 mm (excluding ovipositor). Head black, with metallic green luster; antennae dark brown, F3 paler apically, F4, F6 and clava white. Mesosoma dark brown with metallic sheen; basal half of tegula white, apical half dark brown; wings mostly hyaline but fore wing weakly infuscate at base and with a distinct, broad brown fascia across wing extending from parastigma, marginal and stigmal veins, setae distad of this uniform in color; legs dark brown, except for apical 1/2 of mid basitarsus and mid tarsus 2–4 white. Metasoma black brown.

**Figures 13–18.**
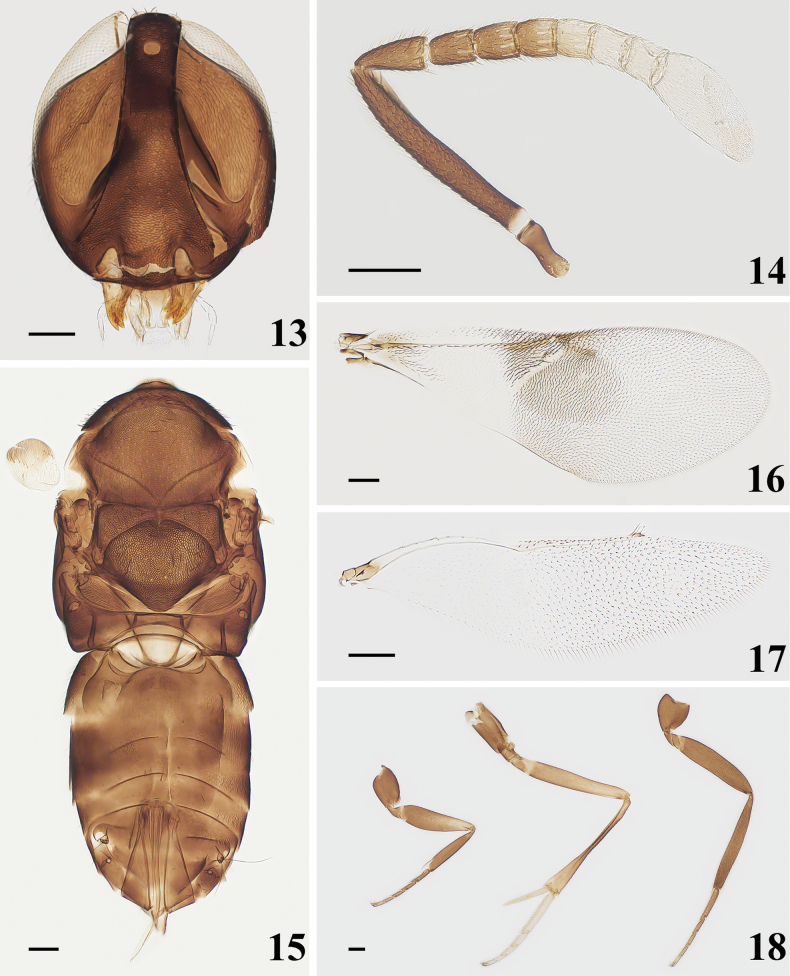
*Homalotylusguangxiensis* Zu, sp. nov., holotype female **13** head **14** antenna **15** mesosoma and metasoma **16** fore wing **17** hind wing **18** legs. Scale bars: 100 μm.

Head (Fig. 13) with sparse setae on frontovertex, each about as long as diameter of posterior ocellus. Head in front view 1.13× higher than wide. Frontovertex 0.2× head width, with distinctly reticulate sculpture. Ocelli forming an angle of about 40°; posterior ocelli very close to eye margin, to occipital margin about 2× as long as the diameter of anterior ocellus. Malar space 0.23× eye height. Antennal torulus with its dorsal margins slightly below lower eye margins, and the distance of antennal torulus by about 1.69× its own height. Antennal (Fig. 14) scape slender, 7.11× as long as wide; pedicel 2.05× as long as wide, 1.63× as long as F1; F1 1.33× as long as wide, F6 slightly wider than long; funicle with linear sensilla on all segments; clava 3-segmented, 2.60× as long as wide, and longer than F4–F6 combined, apex strongly obliquely truncate, truncate part nearly two thirds of the clava. Measurements (μm): HH, 600; HW, 530; FV, 108; EL, 480; MS, 108; OD, 35; POL, 40; OCL, 58; AOL, 88; length and (width)—radicle, 83; scape, 320 (45); pedicel, 98 (48); F1, 60 (45); F2, 58 (50); F3, 53 (50); F4, 53 (50); F5, 53 (55); F6, 50 (60); clava, 163 (63).

Sculpture (Fig. 15) on mesoscutum and scutellum similar to that on head; notaular lines conspicuous and meeting at middle of posterior margin of mesoscutum. Mesoscutum 0.65× as long as wide; scutellum 0.84× as long as wide. Fore wing (Fig. 16) 2.52× as long as wide; linea calva interrupted by 2 lines of setae and closed posteriorly by 3 lines of setae; postmarginal vein about as long as stigmal vein. Hind wing (Fig. 17) 3.68× as long as wide. Length of mid tibial spur (Fig. 18) 0.39× of mid tibia and shorter than mid basitarsus. Measurements (μm): FWL, 1325; FWW, 525; SMV, 560; MV, 53; PMV, 90; SV, 88; HWL, 920; HWW, 250; MT, 740; mid tibial spur, 290; mid basitarsus, 310.

Ovipositor (Fig. 15) 0.76× as long as mid tibia, slightly exserted. Measurements (μm): OL, 560.

**Male.** Unknown.

##### Host.

Unknown.

##### Etymology.

The specific name refers to its collecting location.

##### Diagnosis.

The new species may be distinguished from *H.albitarsus* Gahan, 1910 and *H.agarwali* Anis & Hayat, 1998, by the following characters: F4 completely white (basal brown and apical white in *H.albitarsus*), hind tarsus brown (1–4 white in *H.albitarsus*), mid tibial spur shorter than mid basitarsus (longer in *H.albitarsus*), pedicel 2.05× as long as wide (about 3× in *H.albitarsus*); ovipositor 0.76× as long as mid tibia (1.03× in *H.agarwali*), F3 dark brown, mixed with white apically (white in *H.agarwali*).

#### ﻿ Homalotylus tianjinensis

Taxon classificationAnimaliaHymenopteraEncyrtidae

Zu sp. nov.

709BEEF0-F636-5AD7-B517-5B094513762C

https://zoobank.org/91147952-886C-449A-B0BB-2BFC2C7B889F

Figs 19–25

##### Type materials.

***Holotype*.** ♀ [on slide], China, Tianjin City, Xiqing District, Tianjin Agricultural University, 39°5'21"N, 117°5'38"E, c. 13 m, 18.VI–3.VII.2019, Guo-Hao Zu, Ze-Ning Yang, Malaise trapping. ***Paratypes*.** ♀ [on slide], China, Tianjin City, Xiqing District, Tianjin Agricultural University, 39°5'21"N, 117°5'38"E, c. 13 m, 30.V–14.VI.2020, Ze-Ning Yang, Chen Zhang, Malaise trapping.

##### Description.

**Female.** Holotype. Length, excluding ovipositor, 2.20 mm. Face yellowish brown, frontovertex dark brown, genae dark brown. Antennae dark brown, F6 from brown to white, clava white. Mesosoma black brown, but axilla yellowish brown, scutellum yellowish with a diamond-shaped brown area at the base; tegula white; wings largely hyaline but fore wing infuscate at base and with a distinct, broad more or less parallel-sided brown fascia across immediately distad of this, otherwise apical setae normal and dark; legs dark brown, except for apical 1/5 of mid tibia, all tibial spurs, mid and hind tarsus 1–4 white. Metasoma black, the protruding part of the ovipositor black brown, with only the base yellowish white.

**Figures 19–25.**
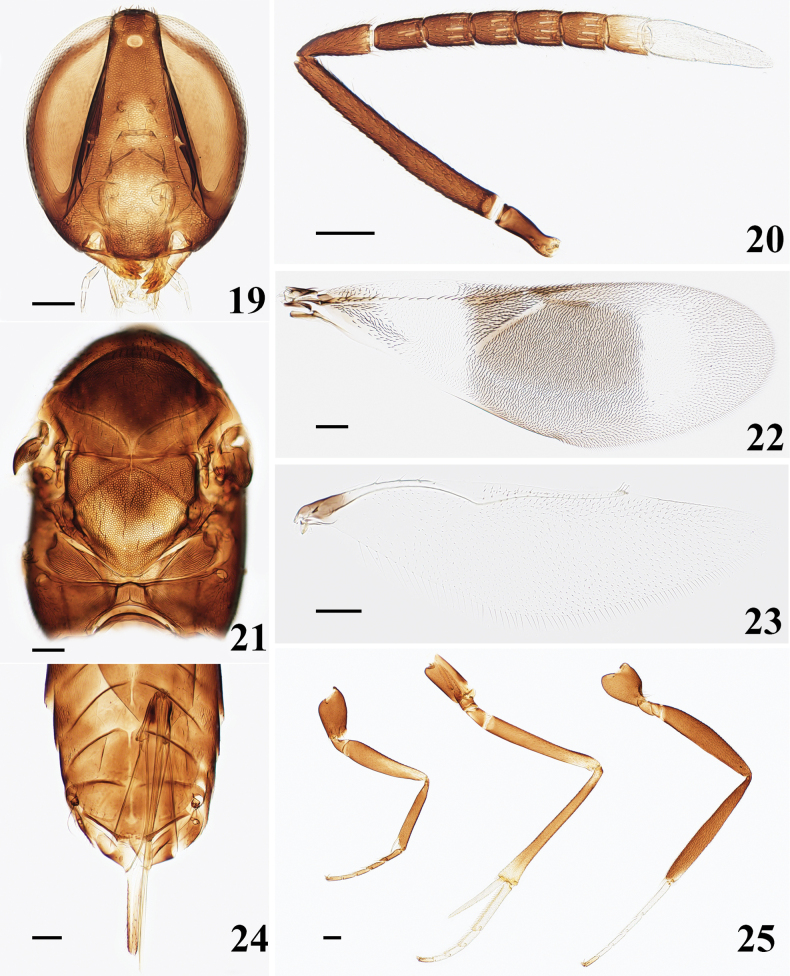
*Homalotylustianjinensis* Zu, sp. nov., holotype, female **19** head **20** antenna **21** mesosoma **22** fore wing **23** hind wing **24** metasoma **25** legs. Scale bars: 100 μm.

Head (Fig. 19) with numerous conspicuous setae on frontovertex, each about as long as diameter of posterior ocellus; piliferous punctures shallow. Head in front view about 1.13× higher than wide. Frontovertex about 1/6 head width, and narrowest about midway between posterior ocelli and occipital margin, eye margins conspicuously diverging anteriorly. Ocelli forming an angle of about 40°. Malar space 0.17× eye height. Antennal torulus with its dorsal margin below lower eye margin and very close to oral margin. Clypeal margin slightly convex. Antennal (Fig. 20) scape slender, almost 7.60× as long as wide; pedicel 2.5× as long as wide, 1.49× as long as F1; all funicle segments longer than wide, F1 1.74× as long as wide, F6 1.26× as long as wide; funicle with linear sensilla on all segments; clava 3-segmented, about as long as F4–F6 combined, apex strongly obliquely truncate, truncate part approach to the middle of the first clava segment. Measurements (μm): HH, 590; HW, 530; FV, 90; EL, 500; MS, 87; OD, 38; POL, 38; OCL, 76; AOL, 66; length and (width)—radicle, 125; scape, 380 (50); pedicel, 130 (52); F1, 87 (50); F2, 85 (57); F3, 80 (60); F4, 82 (62); F5, 80 (62); F6, 82 (65); clava, 232 (65).

Mesosoma (Fig. 21) with sculpture on mesoscutum of similar mesh size to that on frontovertex, but much shallower; sculpture on scutellum similar to that on frontovertex, but slightly deeper and coarser; notaular lines conspicuous but not quite meeting at middle of posterior margin of mesoscutum. Scutellum about as long as wide and about as long as mesoscutum. Fore wing (Fig. 22) 2.78× as long as width; venation and setation as in Fig. 23; costal cell narrow; linea calva interrupted by 3 lines of setae and closed posteriorly by 5 lines of setae; marginal vein about 1.5× as long as wide; postmarginal vein slightly shorter than stigmal vein, angle between them about 30°. Hind wing (Fig. 23) 3.81× as long as width. Length of mid tibial spur (Fig. 25) about 0.45× of mid tibia and longer than mid basitarsus. Measurements (μm): FWL, 1420; FWW, 510; SMV, 645; MV, 62; PMV, 145; SV, 151; HWL, 1030; HWW, 270; MT, 860; mid tibial spur, 390; mid basitarsus, 350.

Ovipositor (Fig. 24) distinctly exserted, 1.14× as long as mid tibia and the exserted part nearly equal to mid tibial spur. Measurements (μm): OL, 980.

**Male.** Unknown.

##### Host.

Unknown.

##### Etymology.

The specific name refers to its collecting location.

##### Diagnosis.

The new species may be distinguished from *H.albiclavatus* (Agarwal, 1970) and *H.vicinus* Silvestri, 1915, by the following characters: scape completely dark brown, 7.60× as long as wide (with a long light-colored strip medially, 8.55× in *H.albiclavatus*), F6 from brown to white (completely white in *H.albiclavatus*), scutellum yellowish with a diamond-shaped brown area at the base (yellow in *H.albiclavatus*); F6 from brown to white (dark in *H.vicinus*), hind femur dark brown (ventrally pale yellow apically in *H.vicinus*), hind tarsus 1–4 white (2–4 white in *H.vicinus*).

#### ﻿ Homalotylus agarwali

Taxon classificationAnimaliaHymenopteraEncyrtidae

Anis & Hayat, 1998

4F58D247-BCC6-567E-B3D1-286C8F19DF3C

Figs 26–31

Homalotylus agarwali Anis & Hayat, 1998: 203–204. Holotype ♀, India, HACO, not examined.

##### Host.

Unknown.

##### Distribution.

China (Guangxi), India.

##### Material examined.

2♀, China, Guangxi Province, Qinzhou City, Beibu Culf University, 21°53'53"N, 108°36'56"E, c. 24 m, 6–17.XI.2019, Wen-quan Zhen, Malaise trapping.

##### Diagnosis.

This is the first record from China.

#### ﻿ Homalotylus hemipterinus

Taxon classificationAnimaliaHymenopteraEncyrtidae

(De Stefani, 1898)

6979A21C-AD75-5638-842D-CB905C12CC00

Figs 32–37

Phaenodiscus hemipterinus De Stefani, 1898: 250. Holotype? ♀, Italy, ? lost.
Homalotvlus orci Girault, 1917a: 3. Syntypes, Indonesia (Java), USNM, examined.
Homalorylus flavimesopleurum Girault, 1917b: 5. Lectotype ♀, Japan, USNM, examined.
Neoaenasioidea nigritus Agarwal, 1970: 27. Holotype ♀, India, ZAMU, not examined.
Echthroplexis tumkurensis Shafee & Fatma, 1985: 375. Holotype ♀, India, ZAMU, not examined.
Homalotvlus evtelweinii (Ratzeburg); Hayat 2006, Trjapitzin and Ruiz Cancino 2003, misidentification.

##### Host.

Coleoptera (Coccinellidae): *Chilocorusbipustulatus*, *Chilocorusbijugus*, *Chilocorusnigritus*, *Chilocorus* sp., *Coccinellaseptempunctata*, *Cyclonedasanguinea*, *Menochilussexmaculatus*.

##### Distribution.

China (Guangxi), Japan, Thailand, India, Myanmar, Malaysia, Indonesia, Iran, Austria, Brazil, Czech Republic, France, Germany, Israel, Italy, Kenya, Congo, Democratic Republic of Congo, Malawi, South Afica, Chad, Sudan, Togo, Cosat Rica, Bahamas, Paraguay, Colombia, Fiji.

##### Material examined.

15♀, China, Guangxi Province, Qinzhou City, Beibu Gulf University, 21°53'53"N, 108°36'56"E, c. 24 m, 26.III–29.XII.2019, Wen-quan Zhen, Malaise trapping.

##### Diagnosis.

This is the first record from China.

**Figures 26–31.**
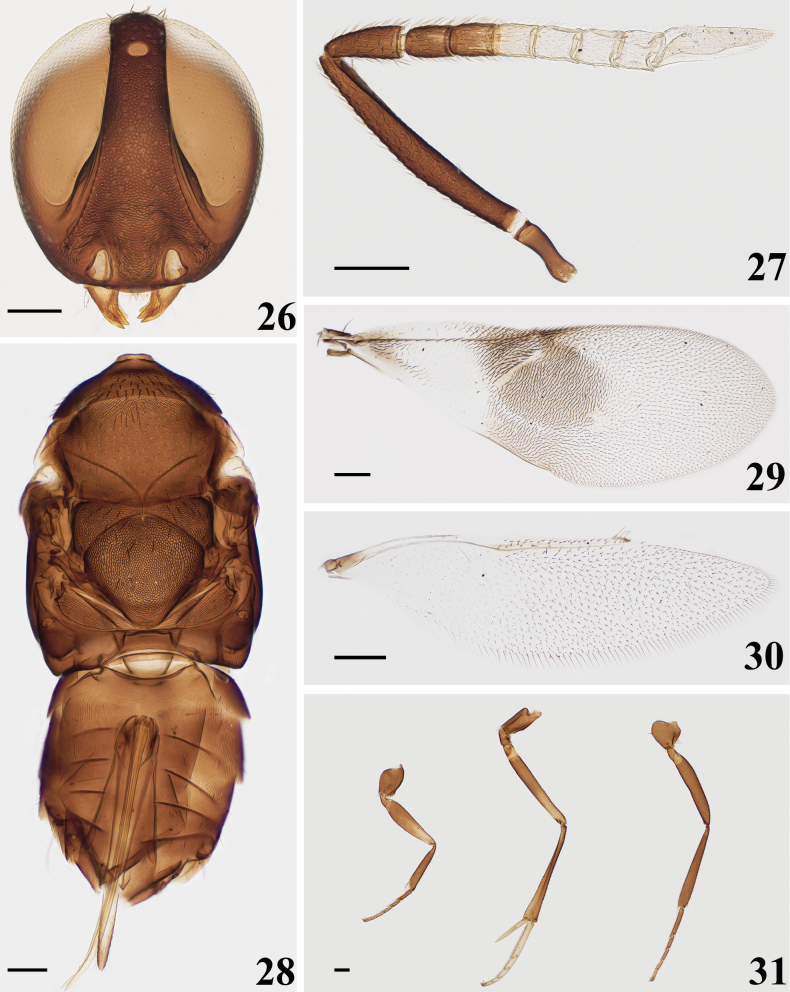
*Homalotylusagarwali* Anis & Hayat, 1998, female **26** head **27** antenna **28** mesosoma and metasoma **29** fore wing **30** hind wing **31** legs. Scale bars: 100 μm.

**Figures 32–37.**
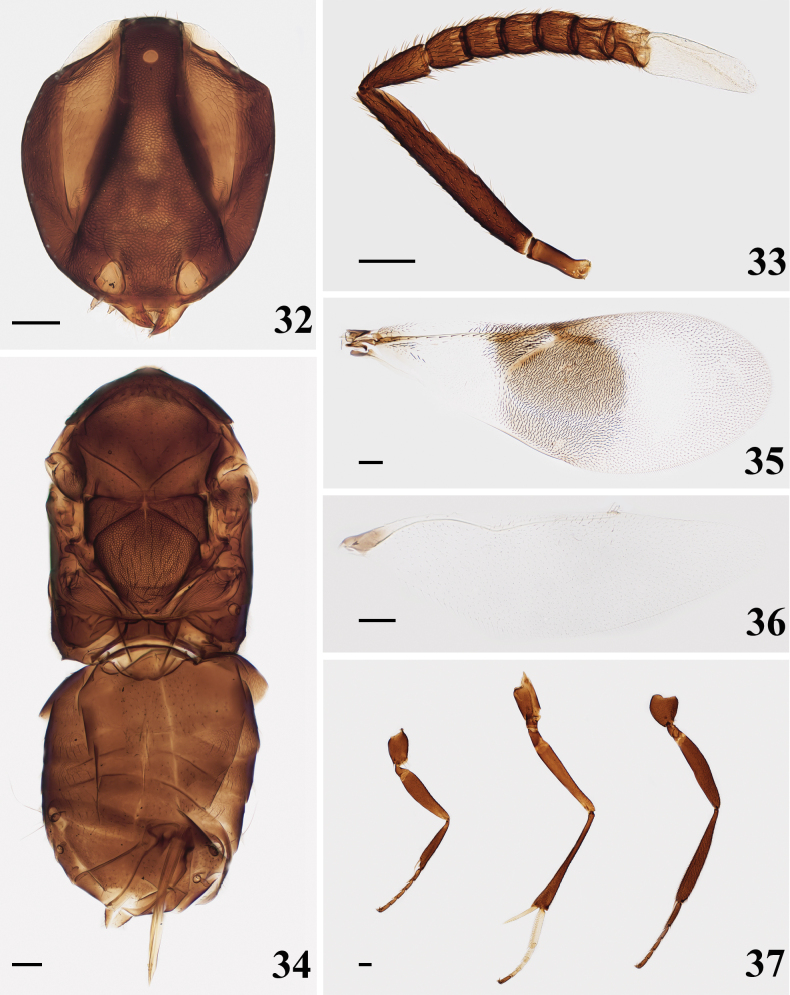
*Homalotylushemipterinus* (De Stefani, 1898), female **32** head **33** antenna **34** mesosoma and metasoma **35** fore wing **36** hind wing **37** legs. Scale bars: 100 μm.

#### ﻿ Homalotylus varicolorus

Taxon classificationAnimaliaHymenopteraEncyrtidae

Krishnachaitanya & Manickavasagam, 2016

99D57E7E-21FA-5C8A-AC35-84F440DB9837

Figs 38–43 , 44–49

Homalotylus varicolorus Krishnachaitanya, Manickavasagam & Abhinav, 2016: 2373, 2380–2382. Holotype ♀, India, EDAU, not examined.

##### Host.

Unknown.

**Figures 38–43.**
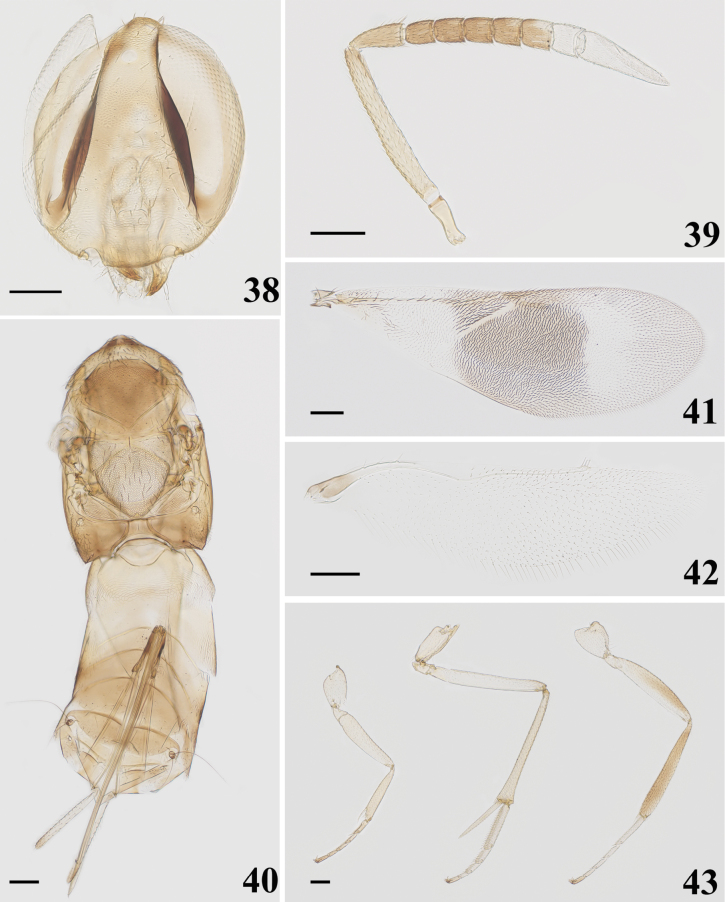
*Homalotylusvaricolorus* Krishnachaitanya & Manickavasagam, 2016, female **38** head **39** antenna **40** mesosoma and metasoma **41** fore wing **42** hind wing **43** legs. Scale bars: 100 μm.

**Figures 44–49.**
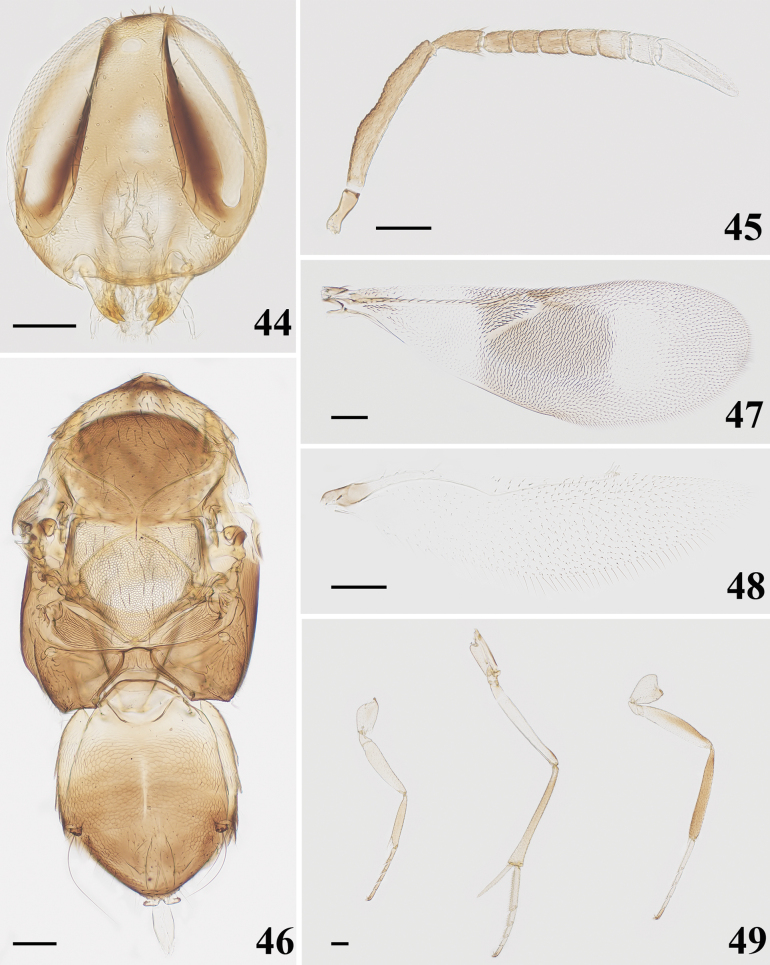
*Homalotylusvaricolorus* Krishnachaitanya & Manickavasagam, 2016, male **44** head **45** antenna **46** mesosoma and metasoma **47** fore wing **48** hind wing **49** legs. Scale bars: 100 μm.

##### Distribution.

China (Guangxi), India.

##### Material examined.

17♀, 16♂, China, Guangxi Province, Qinzhou City, Beibu Gulf University, 21°53'53"N, 108°36'56"E, c. 24 m, 21.IV.2019–19.IV.2020, Wen-quan Zhen, Malaise trapping.

##### Diagnosis.

This is the first record from China. The specimens agree very well with the description of *H.varicolorus*, but a minor difference should be noted. In our specimens ovipositor 1.57× as long as mid tibia, whilst in the original description of *H.varicolorus* ovipositor 2.19× as long as mid tibia.

## Supplementary Material

XML Treatment for Homalotylus bicolor

XML Treatment for Homalotylus guangxiensis

XML Treatment for Homalotylus tianjinensis

XML Treatment for Homalotylus agarwali

XML Treatment for Homalotylus hemipterinus

XML Treatment for Homalotylus varicolorus

## ﻿Additional information

### Author ORCIDs

Guohao Zu https://orcid.org/0000-0002-9892-2171

Hao Xue https://orcid.org/0000-0002-4058-1298

Haiyang Wang https://orcid.org/0009-0007-5665-2111

Wenquan Zhen https://orcid.org/0000-0002-7222-2775

### Data availability

All of the data that support the findings of this study are available in the main text.
